# How pediatric resident’s life has changed during the COVID-19 pandemic

**DOI:** 10.1186/s13052-020-00920-6

**Published:** 2020-10-16

**Authors:** Martina Votto, Maria De Filippo, On behalf of the Pediatric Residents of the University of Pavia, Italy, Amariti Rossella, Andrenacci Beatrice, Apicella Antonia, Bassanese Francesco, Bellanca Emanuela, Bonitatibus Giacomo, Bottino Giovanni, Casari Giulia, Castelli Paola, Cervone Anna, Andrea Martina Clemente, Crapanzano Carmela, Delle Cave Francesco, Di Vincenzo Giulia, Dobbiani Giulia, Donesana Myriam, Fasolino Francesco, Ursula Pia Ferrara, Fiorito Ivan, Garassino Claudia, Gertosio Chiara, Giffoni Francesca, Gregnanin Marco, Guarracino Carmen, Ioimo Irene, Iozzi Lucia, Ippolito Rosario, Landi Enrico, Larocca Anna, Laura Buono, Lavarello Claudio, Anita Leon Joya, Loiacono Francesca, Maggio Alessandra, Mariasol Magistrali, Maglie Erika, Nunzia Pia Manganelli, Michev Alexandre, Moiraghi Alice, Musso Paola, Naso Matteo, Novara Cecilia, Olivero Francesca, Paganelli Valeria, Pagliara Paola, Raffa Giulia, Raviola Chiara, Regalbuto Corrado, Rivano Francesca, Roberto Giulia, Rossi Alessandra, Rossi Federico, Santi Viola, Semeria Mantelli Simona, Silvi Cecilia, Siri Giulia, Spreafico Eugenia, Tondina Enrico, Trabatti Chiara, Veraldi Daniele, Vergori Antonio, Vinci Federica, Vitrani Eleonora, Vittoni Viola, Zecchini Andreana

**Affiliations:** 1 Pediatric Clinic, Fondazione IRCCS Policlinico San Matteo, Pavia, Italy; 2 Department of Clinical, Surgical, Diagnostic and Pediatric Sciences, University of Pavia, Piazzale Golgi, 19, 27100 Pavia, Italy

**Keywords:** Residents, Children, COVID-19 pandemic, Psychophysical wellbeing, Medical education, SARS-CoV-2 infection

## Abstract

Since the World Health Organization declared Coronavirus Disease 2019 (COVID-19) a global pandemic, a few articles were published on the working experience of pediatric residents, especially from the most exposed countries worldwide. Pediatric residents continue to be essential pillars in managing and treating pediatric diseases and are currently fundamental health care providers for every ill patient, including children and adolescents with COVID-19. Although severe acute respiratory syndrome-coronavirus-2 (SARS-CoV-2) infection is changing everyone’s life, this previously unknown disease can represent a training tool and a hard challenge for pediatric residents to improve their skills and take part in an ongoing process of knowledge.

To the Editor,

Since the WHO declared coronavirus disease 2019 (COVID-19) a global pandemic [1], a few articles were published on the working experience of pediatric residents. Italy was one of the most exposed countries worldwide to Severe Acute Respiratory Syndrome-Coronavirus-2 (SARS-CoV-2) infection, and Lombardy was the most affected region [2]. The first case of COVID-19 in Italy was confirmed on February 21, in Pavia. In this context, the University Pediatric Clinic has been promptly involved in the management of the outbreak and was reorganized entirely, involving all medical staff and the sixty-seven residents.

We believe the COVID-19 pandemic has significantly impacted the pediatric residency education and training, and profoundly changed our daily practice.

Firstly, this global pandemic has affected training programs of Italian residents who have the ethical obligation to treat patients, but still need to become specialized and independent physicians. Webinars and online seminars have replaced routine classroom lessons, and the discussion of peculiar clinical cases with tutors was organized in small groups. The urgent need to share experience and transfer knowledge on the management of COVID-19, allowed us to join online updating courses and meetings. Moreover, many of us were also involved in scientific research activities on several clinical aspects of SARS-CoV-2 infection in children and the design of internal protocols for managing chronic pediatric diseases during the pandemic.

To face up the emergency and to limit the risk of infection, residents were split into two teams (Fig. 1). The “clean” team was mainly dedicated to the care of patients hospitalized in Oncology, Neonatal Intensive Care, and Pediatric Inpatients Units. On the other hand, senior residents took part in the “COVID-dedicated” team and worked in the pediatric Emergency Department (ED) that was immediately reorganized with a specific isolation area, and COVID Inpatient Unit. Moreover, some of us voluntarily helped other physicians to take care of adult patients with COVID-19 hospitalized in the Infectious Diseases Department. Besides, starting from the first day of the outbreak in Lombardy, the outpatient services (neurology, cardiology, allergy and immunology, gastroenterology, rheumatology, nephrology, infectious disease) of our Hospital were reorganized and never closed, ensuring urgent examinations and unpostponable procedures. Fortunately, no residents of the “COVID-dedicated” team got sick and tested positive for COVID-19.

**Fig. 1:**
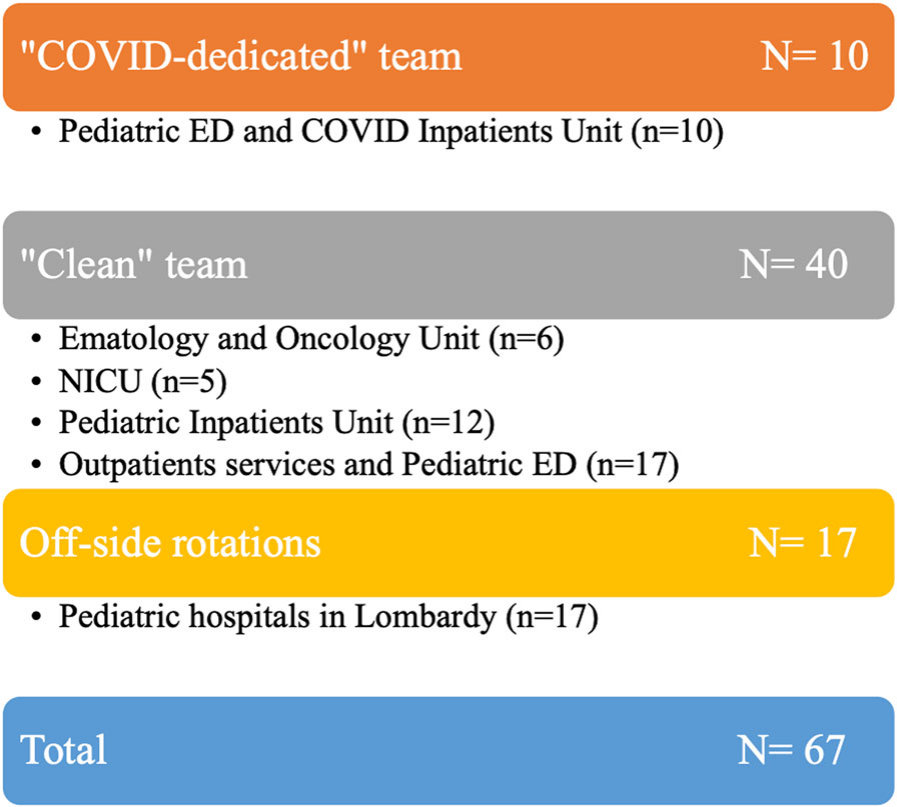
The organization of resident students of the Pediatric Clinic during the COVID-19 pandemic

We attended a worldwide spread of a new infectious disease that even modified the interpretation of prevalent pediatric symptoms. Also, we are dealing with the collective fear of a potential infection, that induced a reduction of admissions in pediatric ED and sometimes diagnostic delay [3]. Although children may develop mild symptoms or be asymptomatic [4], unfortunately, we experienced cases of patients with complicated COVID-19 [5]. The care of children with COVID-19 was not simple. Every day, we got used to visiting scared children with personal protective equipment, playing the part of “an astronaut who got off the stars to visit ill kids.”

In many university hospitals, residents’ psychophysical wellbeing is still uninvestigated and should be assessed, preserved, and eventually supported when mood issues or anxiety appears. Therefore, in our pediatric Clinic, a cohort of 22 residents underwent a psychological test (Beck’s depression inventory) to evaluate the potential development of depressive disorders, particularly in this critical pandemic period. Despite the loneliness and distance from families, the increased healthcare efforts, and the working practice, entirely revolutionized by the pandemic, none of us developed mood issues, and no significant differences in depressive symptoms were reported in “COVID-dedicated” and “clean” team (Table 1). This result may be addressed to the absence of child deaths, the benign course of the infection in most of the affected children, and the limited number of enrolled residents.

**Table 1:** Features of pediatric residents assessed for depression symptoms

	“COVID-dedicated” team	“Clean” team	***p*** value
**Enrolled residents (*n*)**	10	12	
**Male/Female (*n*)**	2/8	2/10	
**Age range (years)**	29–31	27–32	
**BDI total score (mean value)**	4,5	6	0,5
**BDI total score (min-max values)**	0–18	0–15	

*BDI* Beck’s Depression Inventory

Scores: 11–16 mild mood disturbance, 17–20 borderline clinical depression, 21–30 moderate depression, 31–40 severe depression, > 40 extreme depression

During the pandemic, pediatric residents continue to be essential pillars in managing and treating pediatric diseases and are currently fundamental health care providers for every ill patient, including children and adolescents with COVID-19. Although SARS-CoV-2 infection is changing everyone’s life, this previously unknown disease can represent a training tool and a hard challenge for pediatric residents to improve their skills and take part in an ongoing process of knowledge.

## Data Availability

All data generated or analysed during this study are included in this published article.

## Abbreviations

COVID-19: Coronavirus Disease 2019
ED: Emergency Department
NICU: Neonatal Intensive Care Unit
SARS-CoV-2: Severe Acute Respiratory Syndrome-Coronavirus-2

## References

[1] World Health Organization (WHO). WHO director-General's opening remarks at the media briefing on COVID-19; 2020. https://www.who.int/dg/speeches/detail/who-director-general-s-opening-remarks-at-the-media-briefing-on-covid-19%2D%2D-11-march-2020. Accessed 10 June 2020.

[2] WHO Dashboard COVID-19. https://www.who.int/emergencies/diseases/novel-coronavirus-2019. Accessed 10 June 2020.

[3] Lazzerini M, Barbi E, Apicella A, Marchetti F, Cardinale F, Trobia G (2020). Delayed access or provision of care in Italy resulting from fear of COVID-19. Lancet Child Adolesc Health.

[4] Castagnoli R, Votto M, Licari A, Brambilla I, Bruno R, Perlini S, et al. Severe acute respiratory syndrome coronavirus 2 (SARS-CoV-2) infection in children and adolescents: a systematic review. JAMA Pediatr. 2020. 10.1001/jamapediatrics.2020.1467. 10.1001/jamapediatrics.2020.1467 32320004

[5] Brambilla I, Tosca MA, De Filippo M, Licari A, Piccotti E, Marseglia GL, et al. Special issues for COVID-19 in children and adolescents. *Obesity* (Silver Spring). 2020. 10.1002/oby.22878. 10.1002/oby.22878 32396994

